# Confirmed or unconfirmed cases of 2019 novel coronavirus pneumonia in Italian patients: a retrospective analysis of clinical features

**DOI:** 10.1186/s12879-020-05504-7

**Published:** 2020-10-19

**Authors:** Giulia De Angelis, Brunella Posteraro, Federico Biscetti, Gianluca Ianiro, Lorenzo Zileri Dal Verme, Paola Cattani, Francesco Franceschi, Maurizio Sanguinetti, Antonio Gasbarrini

**Affiliations:** 1 Dipartimento di Scienze Biotecnologiche di Base, Cliniche Intensivologiche e Perioperatorie, Università Cattolica del Sacro Cuore, Rome, Italy; 2 Dipartimento di Scienze di Laboratorio e Infettivologiche, Fondazione Policlinico Universitario A. Gemelli IRCCS, Largo A. Gemelli 8, 00168 Rome, Italy; 3 Dipartimento di Scienze Mediche e Chirurgiche, Fondazione Policlinico Universitario A. Gemelli IRCCS, Rome, Italy; 4 Dipartimento di Scienze dell’Emergenza, Anestesiologiche e della Rianimazione, Fondazione Policlinico Universitario A. Gemelli IRCCS, Rome, Italy

**Keywords:** Pneumonia, COVID-19, Clinical and laboratory findings, Outcomes

## Abstract

**Background:**

Since December 2019, the severe acute respiratory syndrome coronavirus 2 (SARS-CoV-2) emerged as a novel etiologic agent of viral pneumonia. We aimed to compare clinical features of 165 Italian patients with laboratory confirmed or unconfirmed 2019-nCoV pneumonia.

**Methods:**

On March 31, 2020, hospitalized patients who presented with fever and/or respiratory symptoms, exposures, and presence of lung imaging features consistent with 2019-nCoV pneumonia were included. Before admission to a hospital ward, patients underwent RT-PCR based SARS-CoV-2 RNA detection in their nasopharyngeal swab samples.

**Results:**

Of 165 patients studied, 119 had positive RT-PCR results and 46 were RT-PCR negative for 2 days or longer (i.e., when the last swab sample was obtained). The median age was 70 years (IQR, 58–78), and 123 (74.6%) of 165 patients had at least one comorbidity. The majority of patients (101/165, 61.2%) had a mild pneumonia, and the remaining patients (64/165, 38.8%) a severe/critical pneumonia. We did not find any substantial difference in symptoms, incubation periods, and radiographic/CT abnormalities as well as in many of the biological abnormalities recorded. However, at multivariable analysis, higher concentrations of hemoglobin (OR, 1.34; 95% CI, 1.11–1.65; *P* = 0.003) and lower counts of leukocytes (OR, 0.81; 95% CI, 0.72–0.90; *P* < 0.001) were statistically associated with confirmed COVID-19 diagnosis. While mortality rates were similar, patients with confirmed diagnosis were more likely to receive antivirals (95% vs 19.6%, *P* < 0.001) and to develop ARDS (63% vs 37%, *P* = 0.003) than those with unconfirmed COVID-19 diagnosis.

**Conclusions:**

Our findings suggest that unconfirmed 2019-nCoV pneumonia cases may be actually COVID-19 cases and that clinicians should be cautious when managing patients with presentations compatible with COVID-19.

## Background

The 2019 novel coronavirus (2019-nCoV), named severe acute respiratory syndrome coronavirus 2 (SARS-CoV-2), became notorious since December 2019 as a new etiologic agent of viral pneumonia [1]. In early illness stages, patients with coronavirus disease 2019 (COVID-19) present with symptoms of acute respiratory infection, which can progress to acute respiratory distress syndrome (ARDS) and other serious complications [2]. Because of substantial pneumonia-related morbidity and mortality [3], testing for SARS-CoV-2 infection of patients who meet the suspected-case definition for COVID-19 [4] is central for their management. Accordingly, provision of supportive care (e.g., oxygenation, ventilation, and fluid therapy) and/or administration of antiviral agents may be decisive [5].

Real-time reverse-transcriptase–polymerase-chain-reaction (RT-PCR) based SARS-CoV-2 RNA detection in respiratory samples (e.g., nasopharyngeal swabs) is the reference diagnostic method to confirm COVID-19 [6]. However, one or more negative results do not exclude the likelihood of SARS-CoV-2 infection [4]. Currently published studies suggest lung imaging, biomarkers, and other non-microbiological tests as ancillary diagnostic methods [6], encouraging further investigation to understand the value of radiological or laboratory findings to diagnose COVID-19. We comparatively explored the clinical features of 165 patients with laboratory confirmed or unconfirmed 2019-nCoV pneumonia admitted to COVID-19 wards of the Fondazione Policlinico A. Gemelli IRCCS, which is a tertiary care university hospital in Rome, Italy. Thus, we investigated the prospect that cases with a negative RT-PCR test result are actually cases of 2019-nCoV pneumonia or, in other words, are not to distinguish from those without confirmation test performed.

## Methods

This retrospective, single-center observational study was conducted in accordance with the Declaration of Helsinki and was approved by the Ethics Committee of the Fondazione Policlinico A. Gemelli IRCCS (reference number 17057/20), and written informed consent was obtained from each enrolled patient. All patients who were hospitalized for suspected 2019-nCoV pneumonia [7] on March 31, 2020 were considered for recruitment. Inclusion criteria were fever and/or respiratory symptoms, exposures, and presence of lung imaging features consistent with 2019-nCoV pneumonia [8]. At the emergency room (before admission to a hospital ward), all patients had undergone nasal and oropharyngeal swabs for detection of one or more SARS-CoV-2 specific nucleic acid targets [9], using the Korean Ministry of Food and Drug Safety approved Allplex 2019-nCoV assay (Arrow Diagnostics S.r.l., Genova, Italy). Samples resulted negative had been repeated after 48–72 h. To rule out the presence of infections due to common viral (adenovirus, coronaviruses 229E, HKU1, NL63, OC43, influenza viruses, rhinovirus/enterovirus, etc.) or bacterial (*Bordetella pertussis*, *Chlamydophila pneumoniae*, *Legionella pneumophila*, *Mycoplasma pneumoniae*) pathogens, patients’ samples had also been tested with the GenMark’s ePlex Respiratory Pathogen Panel assay. Eleven patients yielding positive results for the etiological agents above mentioned were not included.

We retrieved demographic, clinical, laboratory, imaging, treatment, and outcome data from the patients’ medical chart records. We classified cases as mild (see above specification), severe (i.e., dyspnea, respiratory rate ≥ 30 breaths/min, blood oxygen saturation ≤ 93%, and partial pressure of arterial oxygen [PaO_2_]/fraction of inspired oxygen [FiO_2_] ≤300 mmHg), or critical (i.e., respiratory failure, septic shock, and/or multiple organ failure) pneumonia. We recorded chest X-ray or computed tomography (CT) features using the Fleischner Society terminology [10], we defined ARDS based on timing, lung imaging, origin of edema, and oxygenation as specified in the Berlin definition [11], and we defined liver injury as transaminase elevation two- to three-times the upper limit of normal (e.g., 45 U/L for alanine aminotransferase). According to the Italian Society of Infectious Diseases guidelines for COVID-19 treatment [12], we administered antiviral agents (e.g., lopinavir/ritonavir) to all patients with severe/critical disease or to patients with mild disease who had comorbidities including age > 70. Categorical variables were expressed as number with percentage and were compared using the χ^2^ test, and continuous variables were expressed as median with interquartile range (IQR) and were compared using the Mann-Whitney *U*-test. A two-sided *P* value of < 0.05 was considered statistically significant. We performed univariate analysis using the aforementioned tests, and all significant variables (among demographics and baseline characteristics) were included in a multivariable logistic regression model to identify those variables that were statistically associated with confirmed COVID-19 diagnosis. Odds ratio (OR) values with 95% confidence intervals (CIs) were calculated. All analyses were performed with Stata software version 11.1 (StataCorp, College Station, TX, USA).

## Results

On March 31, 2020, 176 patients were hospitalized at our center with a suspicion of 2019-nCoV pneumonia, and 165 patients were finally included. Of them, 119 were confirmed COVID-19 cases based on positive RT-PCR results on nasopharyngeal swabs [4], and 46 were RT-PCR negative for 2 days or longer (i.e., when the last swab sample was obtained).

Table 1 shows demographic and clinical characteristics of 165 patients at baseline. The median age was 70 years (IQR, 58–78), and 113 patients were males. One hundred and twenty-three (74.6%) patients had at least one comorbidity. The most common symptoms at admission were fever (*n* = 155, 93.9%), dyspnea (*n* = 92, 55.8%) and cough (*n* = 77, 46.7%), and the median time from symptom onset to COVID-19 diagnosis was 7 days (IQR, 3–10). Overall, lactate dehydrogenase levels (median value, 289 U/L; IQR, 230–415) and C-reactive protein levels (median value, 74.0 mg/L; IQR, 32.2–139.4) were elevated. One hundred and twenty-seven patients (77%) presented with X-ray signs of ground-glass opacity, and 106 (64.2%) with signs of consolidation. At admission, 101 (61.2%) presented with a mild pneumonia, and the remaining 64 patients (38.8%) with a severe/critical pneumonia.

**Table 1:** Demographics, baseline characteristics, and outcomes of patients diagnosed with 2019-nCoV pneumonia

Variable	Total (*n* = 165)	Confirmed Diagnosis^a^ (*n* = 119)	Unconfirmed Diagnosis^a^ (*n* = 46)	*P* Value	OR (95% CI)^b^
Age (years), median (IQR)	70 (58–78)	68 (58–77)	73.5 (58–85)	0.09	…
Male sex	113 (68.5)	85 (71.4)	28 (60.9)	0.19	…
Pre-existing conditions					
Any	123 (74.6)	86 (72.3)	37 (80.4)	0.28	…
Cardiovascular disease	77 (46.7)	59 (49.6)	18 (39.1)	0.23	…
Connective tissue disease	30 (18.2)	23 (19.3)	7 (15.2)	0.54	…
Nervous system disease	29 (17.6)	18 (15.1)	11 (23.9)	0.18	…
Diabetes	22 (13.3)	15 (12.6)	7 (15.2)	0.66	…
Malignancy	21 (12.7)	12 (10.1)	9 (19.6)	0.10	…
Respiratory system disease	18 (10.9)	12 (10.1)	6 (13.0)	0.59	…
Chronic kidney disease	15 (9.1)	8 (6.7)	7 (15.2)	0.09	…
Immunodeficiency	7 (4.2)	3 (2.5)	4 (8.7)	0.08	…
Chronic liver disease	3 (1.8)	3 (2.5)	0 (0.0)	0.28	…
Other^c^	29 (17.6)	17 (14.3)	12 (26.1)	0.07	…
Symptoms at admission					
Fever	155 (93.9)	115 (96.6)	40 (87.0)	0.02	…
Shortness of breath (or dyspnea)	92 (55.8)	63 (52.9)	29 (63.0)	0.24	…
Cough	77 (46.7)	60 (50.4)	17 (37.0)	0.12	…
Diarrhea/Nausea/Vomiting	6 (3.6)	3 (2.5)	3 (6.5)	0.22	…
Days from symptom onset, median (IQR)	7 (3–10)	7 (3–10)	7 (3–10)	0.41	…
Signs at admission, median (IQR)					
Heart rate (beats/min)	89 (80–101)	89 (80–102)	92 (80–100)	0.99	…
Respiration rate (breaths/min)	14 (12–15)	14 (12–16)	13 (12–15)	0.24	…
Blood oxygen saturation (%)	94 (90–96)	94 (90–96)	93 (90–97)	0.74	…
Systolic blood pressure (mmHg)	130 (120–140)	130 (120–140)	130 (118–140)	0.97	…
Diastolic blood pressure (mmHg)	80 (70–86)	80 (70–86)	75 (66–88)	0.56	…
qSOFA score (≥2)	25 (15.2)	17 (14.3)	8 (17.4)	0.62	…
Chest X-ray/CT findings					
Ground-glass opacity (GGO)	127 (77.0)	96 (80.7)	31 (67.4)	0.07	…
Consolidation	106 (64.2)	79 (66.4)	27 (58.7)	0.35	…
Pleural effusion	40 (24.2)	24 (20.2)	16 (34.8)	0.19	…
CT findings only^d^					
GGO and reticular	29/112 (17.6)	21/76 (17.6)	8/36 (17.4)	0.54	…
Pleural thickening/retraction	6/112 (3.6)	3/76 (2.5)	3/36 (6.5)	0.34	…
Fibrotic steaks	5/112 (3.0)	3/76 (2.5)	2/36 (4.3)	0.70	…
Air bronchogram	3/112 (1.8)	2/76 (1.7)	1/36 (2.2)	0.96	…
Bronchus distortion	2/112 (1.2)	2/76 (1.7)	0/36 (0.0)	0.33	…
Spectrum of disease					
Mild	101 (61.2)	72 (60.5)	29 (63.0)	0.76	…
Severe	53 (32.1)	39 (32.8)	14 (30.4)	0.77	…
Critical	11 (6.7)	8 (6.7)	3 (6.5)	0.96	…
Blood parameters, median (IQR)					
Leucocytes (×10^9^/L; normal range 4.0–10.0)	6.4 (4.8–9.7)	6.0 (4.7–8.1)	10.1 (6.1–15.4)	< 0.001	0.81 (0.72–0.90)
Neutrophils (×10^9^/L; normal range 2.0–7.0)	4.9 (3.5–7.7)	4.6 (3.3–6.3)	7.7 (4.6–11.7)	< 0.001	…
Lymphocytes (×10^9^/L; normal range 1.0–3.0)	1.1 (0.8–1.4)	1.1 (0.8–1.4)	1.2 (0.7–1.6)	0.78	…
Neutrophils (%)	77.1 (69.5–82.8)	76.3 (67.6–82.4)	80.1 (71.9–87.3)	0.03	…
Lymphocytes (%)	15.8 (10.5–23.4)	17.6 (12.5–24.3)	12.1 (7.6–20.4)	0.003	…
Platelets (×10^9^/L; normal range 150–450)	210 (169–264)	203 (165–252)	250 (189–301)	0.01	…
Hemoglobin (g/dL; normal range 13–17)	13.8 (12.1–14.9)	14.1 (13.0–15.1)	12.1 (10.6–14.1)	< 0.001	1.34 (1.11–1.65)
Serum parameters, median (IQR)					
Alanine aminotransferase (U/L; normal range 7–45)	24 (16–39)	26 (17–40)	19 (13–33)	0.02	…
Lactate dehydrogenase (U/L; normal range 120–250)	289 (230–415)	316 (254–433)	245 (195–336)	< 0.001	1.00 (0.99–1.01)
Creatinine (mg/dL; normal range: 0.7–1.1)	0.9 (0.8–1.3)	0.9 (0.8–1.3)	1.0 (0.8–1.3)	0.59	…
Creatine kinase (U/L; normal range 30–170)	120 (66–219)	122 (72–218)	107 (51–219)	0.36	…
Urea (mg/dL; normal range 10–23)	17 (14–26)	17 (13–25)	19 (15–32)	0.32	…
Infection-related biomarkers, median (IQR)					
Procalcitonin (ng/mL; normal range 0–0.5)	0.09 (0.04–0.18)	0.08 (0.04–0.15)	0.15 (0.07–0.63)	0.006	…
C-reactive protein (mg/L; normal range 0–5.0)	74.0 (32.2–139.4)	66.9 (33.6–130.5)	93.6 (22.1–163.9)	0.59	…
Complications					
Acute respiratory distress syndrome	92 (55.8)	75 (63.0)	17 (37.0)	0.003	…^e^
Liver injury	47 (28.5)	38 (31.9)	9 (19.6)	0.11	…^e^
Septic shock	14 (8.5)	7 (5.9)	7 (15.2)	0.06	…^e^
Admission to intensive care unit	14 (8.5)	12 (10.1)	2 (4.4)	0.23	…^e^
Treatment					
Oxygen support (nasal cannula)	128 (77.6)	98 (82.4)	30 (65.2)	0.02	…^e^
Antiviral therapy	122 (73.9)	113 (95.0)	9 (19.6)	< 0.001	…^e^
Antibiotic therapy	133 (80.6)	93 (78.2)	40 (87.0)	0.20	…^e^
Interleukin*-*6 receptor inhibitor therapy	46 (27.9)	45 (37.8)	1 (2.2)	< 0.001	…^e^
Outcome					
Discharged	125 (75.8)	88 (74.0)	37 (80.4)	0.38	…^e^
Still in hospital as of 4/23/2020^f^	24 (14.6)	20 (16.8)	4 (8.7)	0.18	…^e^
Died	16 (9.7)	11 (9.2)	5 (10.9)	0.75	…^e^

Data are no. (%) unless specified otherwise. Denominators indicate data lacking for 53 patients. Abbreviations: OR, Odds ratio; CI, confidence interval; IQR, interquartile range; qSOFA, quick sepsis-related organ failure assessment; CT, computed tomography

^a^Laboratory-based confirmation of 2019-nCoV pneumonia was done by SARS-CoV-2 RNA detection using a well-established RT-PCR assay [9]

^b^According to multivariable logistic regression model analysis (see text for details)

^c^Includes anemia, endocrine disorders, inflammatory bowel disease, and obesity

^d^CT findings in all 112 patients were assessed according to imaging features described elsewhere [10]

^e^Not calculated because the relative variable was not entered into multivariable logistic regression model (see text for details)

^f^Patients were admitted between 3/6/2020 and 3/31/2020, with follow-up through 4/23/2020

Treatments and outcomes of 165 patients are detailed in Table 1. Overall, 92 patients (55.8%) developed ARDS, and 14 of them (8.5%) septic shock, which needed transfer to ICU. Most patients (*n* = 128, 77.6%) were treated with oxygen support, antivirals (*n* = 122, 73.9%), and antibiotics (*n* = 133, 80.6%). Forty-six patients (27.9%) received therapy with interleukin-6 receptor inhibitors. Overall, 16 (9.7%) of 165 patients died at the follow-up end (*n* = 13 because ARDS, *n* = 2 because of septic shock, *n* = 1 because of multiple comorbidities).

At univariate analysis, fever was significantly more frequent in patients with confirmed diagnosis (96.6% vs 87.0%, *P* = 0.02). This group presented also with significantly lower levels of leucocytes (median value, 6.0 × 10^9^/L vs 10.1 × 10^9^/L; *P* < 0.001), neutrophils (median value, 4.6 × 10^9^/L vs 7.7 × 10^9^/L; *P* < 0.001), platelets (median value, 203 × 10^9^/L vs 250 × 10^9^/L; *P* = 0.01), and procalcitonin (median value, 0.08 ng/mL vs 0.15 ng/mL, *P* = 0.006), and higher levels of hemoglobin (median value, 14.1 g/dL vs 12.1 g/dL; *P* < 0.001), alanine aminotransferase (median value, 26 U/L vs 19 U/L, *P* = 0.02), and lactate dehydrogenase (median value, 316 U/L vs 245 U/L; *P* < 0.001). Patients with confirmed diagnosis were also more likely to receive antivirals (95% vs 19.6%, *P* < 0.001) and to develop ARDS (63% vs 37%, *P* = 0.003) than those without confirmed diagnosis. There were no other significant differences between the two groups.

At multivariable analysis, higher concentrations of hemoglobin (OR, 1.34; 95% CI, 1.11–1.65; *P* = 0.003) and lower counts of leukocytes (OR, 0.81; 95% CI, 0.72–0.90; *P* < 0.001) were found to be statistically associated with confirmed diagnosis in the overall cohort.

## Discussion

We tested the hypothesis that negative patients did not differ from SARS-CoV-2 RNA positive patients by comparing features of 165 cases with clinically diagnosed 2019-nCoV pneumonia in our hospital. We did not find any substantial difference in symptoms, incubation periods, and radiographic/CT abnormalities as well as in many of the biological abnormalities recorded. However, blood/serum test results showed that patients with laboratory-confirmed diagnosis of 2019-nCoV pneumonia were more likely to have higher levels of hemoglobin and lower levels of leukocytes. Additionally, the proportion of ARDS in the group of COVID-19 confirmed patients was significantly higher than in the group of COVID-19 unconfirmed patients.

Large or small descriptive studies published in 2020 mainly focused on patients with laboratory-confirmed 2019-nCoV pneumonia [2, 13, 14]. Nonetheless, among 72,314 cases (as of February 11, 2020) from the COVID-19 outbreak in China, 16,186 (22%) and 10,567 (15%) of them could not receive laboratory confirmation for COVID-19 and, then, were classified as suspected cases or clinically diagnosed cases, respectively [8]. Although testing for SARS-CoV-2 in our laboratory was not restricted [15], 46 (27.8%) of 165 patients with 2019-nCoV pneumonia did not have a laboratory-confirmed diagnosis in our study. Despite a well-documented active virus replication in the upper respiratory tract [16], swab samples may have a limited sensitivity to identify cases. Sampling or testing related factors may be responsible for false-negative RT-PCR results, necessitating additional sample collection from the lower respiratory tract specimens including sputum [4].

It is plausible that our SARS-CoV-2 negative patients were outside the window of peak shedding in the upper respiratory tract samples or did not have symptoms highly suggestive for upper respiratory tract infection [16]. Accordingly, eight of 119 patients with positive RT-PCR results became positive only with swabs taken on subsequent days after the first (negative) sampled swab, and their median time from symptom onset did not differ from that of 111 remaining patients (7 days vs 7 days, *P* = 0.76). Furthermore, the sensitivity of the Allplex 2019-nCoV assay might have limited by its requirement that three genes were all detectable for a positive result. In our study, all 46 patients had a radiological evidence of pneumonia not attributed to any typical respiratory viral infection agents, including the human coronaviruses HKU1, OC43, NL63, 229E, the influenza virus A and B, and others (data not shown). It is worthy to note that the finding of typical ground glass opacities in chest CTs of clinically diagnosed patients [17] prompted the Chinese authorities, at one point in early February 2020, to count these patients as confirmed cases [18]. This allowed identifying and quarantining patients as early as possible.

In conclusion, using the clinical diagnosis as the reference standard, the RT-PCR testing allowed to correctly identify two thirds of our patients as COVID-19 while one third was not correctly identified. However, as undocumented SARS-CoV-2 infections may be a relevant source of transmission among hospitalized patients [19], we believe that clinicians should exceed on the side of caution when managing patients with presentations compatible with COVID-19.

## Data Availability

The datasets generated and analyzed during the current study are not publicly available as the data also forms part of an ongoing study but are available from the corresponding author on reasonable request.

## Abbreviations

2019-nCoV: 2019 novel coronavirus
SARS-CoV-2: Severe acute respiratory syndrome coronavirus 2
COVID-19: Coronavirus disease 2019
ARDS: Acute respiratory distress syndrome
RT-PCR: Reverse-transcriptase–polymerase-chain-reaction
PaO_2_: Partial pressure of arterial oxygen
FiO_2_: Fraction of inspired oxygen
CT: Computed tomography
IQR: Interquartile range
OR: Odds ratio
CI: Confidence interval
ICU: Intensive care unit
